# Gut Dysbiosis, Bacterial Colonization and Translocation, and Neonatal Sepsis in Very-Low-Birth-Weight Preterm Infants

**DOI:** 10.3389/fmicb.2021.746111

**Published:** 2021-10-07

**Authors:** Chien-Chung Lee, Ye Feng, Yuan-Ming Yeh, Reyin Lien, Chyi-Liang Chen, Ying-Li Zhou, Cheng-Hsun Chiu

**Affiliations:** ^1^Division of Neonatology, Department of Pediatrics, Chang Gung Memorial Hospital, Chang Gung University College of Medicine, Taoyuan, Taiwan; ^2^Sir Run Run Shaw Hospital, Institute for Translational Medicine, Zhejiang University School of Medicine, Hangzhou, China; ^3^Genomic Medicine Core Laboratory, Chang Gung Memorial Hospital, Taoyuan, Taiwan; ^4^Molecular Infectious Disease Research Center, Chang Gung Memorial Hospital, Taoyuan, Taiwan; ^5^Division of Pediatric Infectious Diseases, Department of Pediatrics, Chang Gung Memorial Hospital, Chang Gung University College of Medicine, Taoyuan, Taiwan

**Keywords:** gut, dysbiosis, microbiota, sepsis, very-low-birth-weight, preterm infants

## Abstract

Gut dysbiosis may precede neonatal sepsis, but the association is still not well-understood. The goal of this study is to investigate the association between gut microbiota and neonatal sepsis, and to seek the evidence of colonization of pathogenic bacteria in the gut before evolving into an invasive infection. A prospective cohort study examined fecal microbiota composition in preterm infants with and without sepsis. Thirty-two very-low-birth-weight (VLBW) preterm infants and 10 healthy term infants as controls were enrolled. The fecal samples collected from the participants at the first, fourth, and seventh weeks of life underwent 16S rRNA amplicon sequencing for measurement of the diversity and composition of the microbiota. The bacterial isolates causing neonatal sepsis were genome sequenced. PCR was performed to confirm the translocation of the bacteria from the gut to the blood. The results showed that VLBW preterm infants with sepsis had lower microbial diversity in the gut at birth compared to preterm infants without sepsis and term infants. The composition of gut microbiome in preterm infants was similar to healthy terms at birth but evolved toward dysbiosis with increasing *Proteobacteria* and decreasing *Firmicutes* weeks later. The strain-specific PCR confirmed the presence of causative pathogens in the gut in 4 (40%) out of 10 VLBW preterms with sepsis before or at onset of sepsis, and persistence of the colonization for weeks after antibiotic treatment. The same bacterial strain could horizontally spread to cause infection in other infants. Prolonged antibiotic exposure significantly reduced beneficial *Bifidobacterium* and *Lactobacillus* in the gut. In conclusion, preterm infants with gut dysbiosis are at risk for neonatal sepsis, and the causative pathogens may be from the gut and persist to spread horizontally. The association of increased *Proteobacteria* abundance and decrease in microbiome diversity suggests the need for interventions targeting the gut microbiome to prevent dysbiosis and sepsis in VLBW preterm infants.

## Introduction

Neonatal sepsis remains an important cause of mortality and long-term morbidity among infants, especially for very-low-birth-weight (VLBW) infants in neonatal intensive care units (NICUs) (van Vliet et al., 2013; Shah and Padbury, 2014). According to the time and mode of infection, neonatal sepsis has been classified as either early-onset sepsis (EOS), late-onset sepsis (LOS), or very late-onset sepsis (VLOS) (Camacho-Gonzalez et al., 2013; Cantey et al., 2014; Bartlett et al., 2017; Shane et al., 2017). EOS is defined as the infections occurring in the first 72 h of life and is acquired before or during delivery due to vertical mother-to-infant transmission. LOS and VLOS are infections appearing between 4 and 90 days of life, respectively, usually among preterm infants with prolonged hospitalization (Guilbert et al., 2010; Cantey et al., 2014; Bartlett et al., 2017). Unlike EOS that has been well-characterized, the origins and mechanisms of LOS and VLOS are not fully understood and can be acquired horizontally from hospital environments or community (Camacho-Gonzalez et al., 2013; Simonsen et al., 2014; Wynn et al., 2014; Cortese et al., 2016; Shane et al., 2017), or from maternal vertical transmission (Vaciloto et al., 2002; Simonsen et al., 2014).

The gut where microbiota constitutes the most abundant microbial community is a natural reservoir for pathogens in neonates (Basu, 2015). Many studies have demonstrated that the development of gut microbiota in infants can be perturbed by many factors (Penders et al., 2006; Bokulich et al., 2016), including the mode of delivery (Huurre et al., 2008; Dominguez-Bello et al., 2010; Azad et al., 2013), antibiotics exposure (Westerbeek et al., 2006; Jernberg et al., 2007; Fouhy et al., 2012; Mazzola et al., 2016; Schwartz et al., 2020), gestational age (Groer et al., 2014; La Rosa et al., 2014; Bäckhed et al., 2015; Korpela et al., 2018), and diet (Harmsen et al., 2000; Penders et al., 2005; Azad et al., 2013; Pannaraj et al., 2017). However, the association between gut microbiota and neonatal sepsis is still not well-understood. While a few studies indicated that intestinal dysbiosis may precede the development of neonatal sepsis (Madan et al., 2012; Mai et al., 2013; Taft et al., 2015; Alverdy and Krezalek, 2017), there is insufficient information about the mechanism of gut dysbiosis leading to sepsis or what key components of healthy microbiota to prevent infections. We therefore performed a prospective observational study on VLBW preterm infants who are at risk of neonatal sepsis. The 16S rRNA sequencing of gut microbiota and genomic analysis of bacterial isolates were carried out to investigate the association between the gut microbiota and neonatal sepsis and to seek the evidence of bacterial pathogens colonizing in the gut before evolving to invasive infections.

## Results

### Clinical Characteristics of Study Participants

From August 2018 to October 2020, 44 VLBW preterm infants were signed in for this study. We excluded 10 infants who had no stool passage within the first postnatal week, one infant who missed the third stool collection time, and one who died of pneumothorax with pulmonary hypertension after second stool collection. Finally, 32 VLBW infants and 10 healthy term infants were enrolled. Ten preterm infants developed 12 episodes of invasive bacterial infections, with their fecal samples being classified as the preterm invasive group (PI). The other fecal samples from 22 preterm infants without invasive infections were designated as the preterm non-invasive group (PNI). Stools collected from term infants were classified as the healthy term group (HT). The clinical information is summarized in Table 1. The mean gestational age of the preterm infants in the PI group was younger, relative to the preterms in the PNI group (26.9 ± 2.2 vs. 28.7 ± 2, *p* = 0.027), but the average birth weight was similar between the two groups (1021.6 ± 283.4 vs. 1155.8 ± 161.4, *p* = 0.188). Infants with invasive bacterial infections also had less cumulative human milk, formula milk, and total feeding consumptions during the first week, but only formula milk was statistically significant (Table 1). The duration of empiric antibiotic use and proportion of probiotic use in the first week was similar between the PI and PNI groups; the DOTs until the third stool collection time (7 weeks) were also not significantly different between the two groups (Table 1). The duration of hospitalization was longer for the PI group, but the difference was not statistically significant between the two groups (Table 1).

**TABLE 1:** Clinical information of very-low-birth-weight (VLBW) preterm infants with or without neonatal sepsis.

	**Preterm infants with sepsis *N* = 10**	**Preterm infants without sepsis *N* = 22**	***P-*value**
Gestational age (weeks)^a^	26.9 ± 2.2	28.7 ± 2.0	0.027
Birth weight (g)^a^	1021.6 ± 283.4	1155.8 ± 161.4	0.188
Cesarean section, n (%)	6 (60.0)	16 (72.7)	0.683
1st week cHM (ml/kg)^a^	5.2 ± 5.0	23 ± 41.8	0.194
1st week cRF (ml/kg)^a^	3.9 ± 2.6	26.6 ± 49.8	0.045
1st week cFeeding (ml/kg)^a^	9.1 ± 5.5	49.6 ± 80.7	0.127
1st week Abx (days)^a^	5.1 ± 1.9	4.9 ± 1.7	0.775
1st week Probiotics, n (%)	4 (40.0)	12 (54.5)	0.446
Antacid use, n (%)	5 (50.0)	5 (22.7)	0.217
DOTs till 3rd stool collection^b^	27.5 (IQR: 18–57)	19.5 (IQR: 8–26)	0.095
Hospitalization days^b^	111.5 (IQR: 68–133)	67.0 (IQR: 53–83)	0.084

*^a^Presented as mean ± standard deviation.*

*^b^Presented as median and interquartile range, IQR.*

*1st week cHM: cumulative human milk consumption within first week.*

*1st week cRF: cumulative formula milk consumption within first week.*

*1st week cFeeding: cumulative feeding within first week.*

*1st week Abx: cumulative antibiotics days within first week.*

*DOTs: Duration of therapy for all antibiotic use (days).*

The bacterial isolates of sepsis in these preterm infants are listed in Table 2. Nine had bloodstream infection, and three had central nervous system (CNS) infection. These infections were caused by Gram-negative bacilli (GNB) in three (25.0%), Gram-positive bacilli (GPB) in one (8.3%), and Gram-positive cocci (GPC) in eight (67.7%). All GPC were *Staphylococcus* species. Patient C06 experienced two invasive infections: one by *Staphylococcus capitis* at 27 days of age and the other by carbapenem-resistant *Serratia liquefaciens* at 48 days of age. Another patient C38 had hydrocephalus with ventriculo-peritoneal shunt (VPS) implantation and developed two distinct invasive CNS infections: one by *Staphylococcus epidermidis* sequence type (ST)35 at 95 days of age and the other by *S. epidermidis* ST59 at 116 days of age. The MLST analysis revealed that infection in patient C03 was also caused by *S. epidermidis* ST35, and infection in patient C28 was caused by *S. epidermidis* ST59. Based on the time of onset, the 12 episodes of invasive infections included 1 EOS, 8 LOS, and 3 VLOS.

**TABLE 2:** Bacterial pathogens causing 12 episodes of neonatal sepsis in 10 preterm infants, the intensive care measures used, and the proportions of bacterial genus identical to the causative pathogens in gut microbiota.

**Case**	**Pathogens (source)**	**Genotype**	**Intensive care measures**			**Age (days)**	**Genus proportions in stool**		
			**Catheter**	**Intravenous feeding**	**Ventilator**		**1-week (%)**	**4-week (%)**	**7-week (%)**
C08	*Klebsiella pneumoniae* (B)	ST1296	PCVC	NPN, lipid	NIV	33	43.9	82.4	93.3
C44	*Escherichia coli* (B)	ST1193	No catheter	No use	NIV	0	99.8	1.8	44.9
C06-2	*Serratia liquefaciens* (B)	NA	PCVC	NPN	IMV	47	0.8	0.1	0.7
C42	*Bacillus cereus* (B)	ST427	PCVC	NPN, lipid	IMV	24	0	0	0
C06-1	*Staphylococcus capitis* (B)	NA	PCVC	NPN, lipid	HFOV	27	8.2	4.2	0.3
C03	*Staphylococcus epidermidis* (B)	ST35	No catheter	No use	Room air	98	0	0	0.94
C19	*Staphylococcus capitis* (B)	NA	PCVC	TPN	HFOV	25	0	<0.1	0
C22	*Staphylococcus aureus* (CSF)	ST15	EVD	No use	IMV	70	0.3	0.3	2.4
C28	*Staphylococcus epidermidis* (B)	ST59	PCVC	NPN	IMV	52	93.4	1.1	0.1
C34	*Staphylococcus capitis* (B)	NA	PCVC	NPN, lipid	NIV	36	0	1.9	0.1
C38-1	*Staphylococcus epidermidis* (CSF)	ST35	VPS	No use	Air cannula	94	0	0.1	<0.1
C38-2	*Staphylococcus epidermidis* (CSF)	ST59	VPS	No use	Room air	116	43.9	82.4	93.3

*B, Blood; CSF, cerebrospinal fluid; EVD, external ventricular drainage; GNB, gram negative bacillus; GPB, Gram positive bacillus; GPC, Gram positive coccus; HFOV, High-frequency oscillatory ventilation; IMV, Intermittent mechanical ventilation; NA, not available; NIV, Non-invasive ventilation; NPN, neonatal parenteral nutrition; PCVC, peripheral central venous catheter; ST, sequence type; VPS, ventriculo-peritoneal shunt.*

### Association Between Gut Dysbiosis and Neonatal Sepsis

**FIGURE 1:**
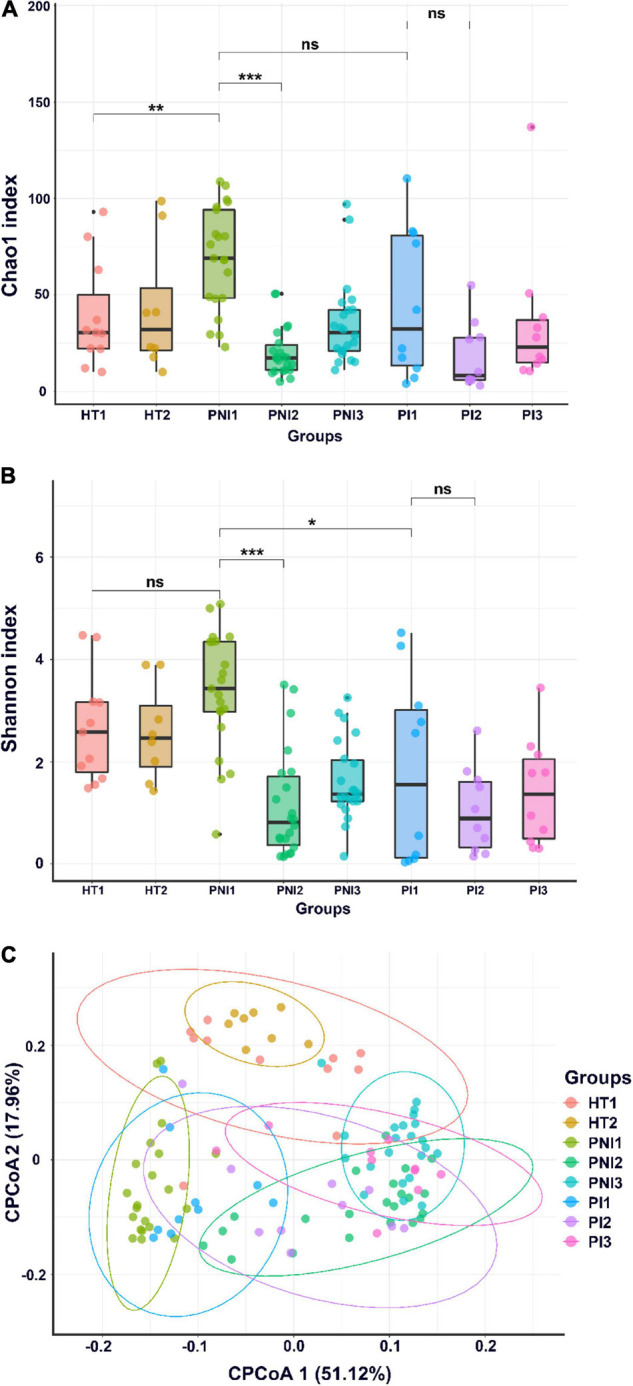
α-diversity and β-diversity of gut microbiota. α-diversity indices, including Chao1 **(A)** and Shannon index **(B)**, and **(C)** β-diversity (CPCoA, covariate adjusted principal coordinates analysis) of gut microbiota of healthy term infants (HT), preterm infants without invasive infections (PNI), and preterm infants with invasive infections (PI). HT1, stool collected within the first week of age; PNI1 and PI1, stool collected within the first week of age; HT2, PNI2, and PI2, stool collected at 4 weeks of age; PNI3 and PI3, stool collected at 7 weeks of age. *p < 0.05; **p < 0.01; ***p < 0.001; ns, non-significant.

A total of 20 stool samples collected from healthy term infants and 96 from VLBW infants were subject to analysis. Figure 1 shows α- and β-diversity among the three groups. According to the Shannon index, the infants with invasive infection (PI group) had significant lower α-diversity (*p* < 0.05) since birth and persisted to 4 and 7 weeks of age (Figure 1B). According to the Chao1 and Shannon index, the dysbiosis persisted to 7 weeks of life (Figures 1A,B). Preterm infants without invasive infections (PNI group) showed higher microbial diversity in the gut at birth, but the diversity decreased significantly in the following weeks (*p* < 0.001). Healthy terms (HT group) maintained higher diversity at birth and thereafter (Figures 1A,B). The analysis of β-diversity (covariate adjusted principal coordinates analysis) showed significant variations among each group (*p* < 0.001) (Figure 1C). In terms of the dynamic change overtime, we found that microbiota in the HT group remained stable from birth to 4 weeks of age (Figure 1C, two circles in the upper part), but that of the PI and PNI groups changed with age from birth to 7 weeks of age (Figure 1C, the representative circles moved from the left lower part to the right lower part with time).

**FIGURE 2:**
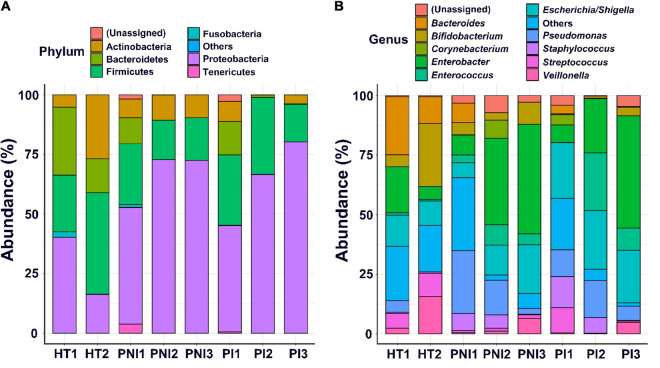
Abundances of bacterial taxa in different groups of the infants. Mean relative abundances of bacterial taxa at phylum **(A)** and genus **(B)** levels in each group of the stool collections. HT, healthy term infants; PNI, preterm infants without invasive infections; PI, preterm infants with invasive infections.

**FIGURE 3:**
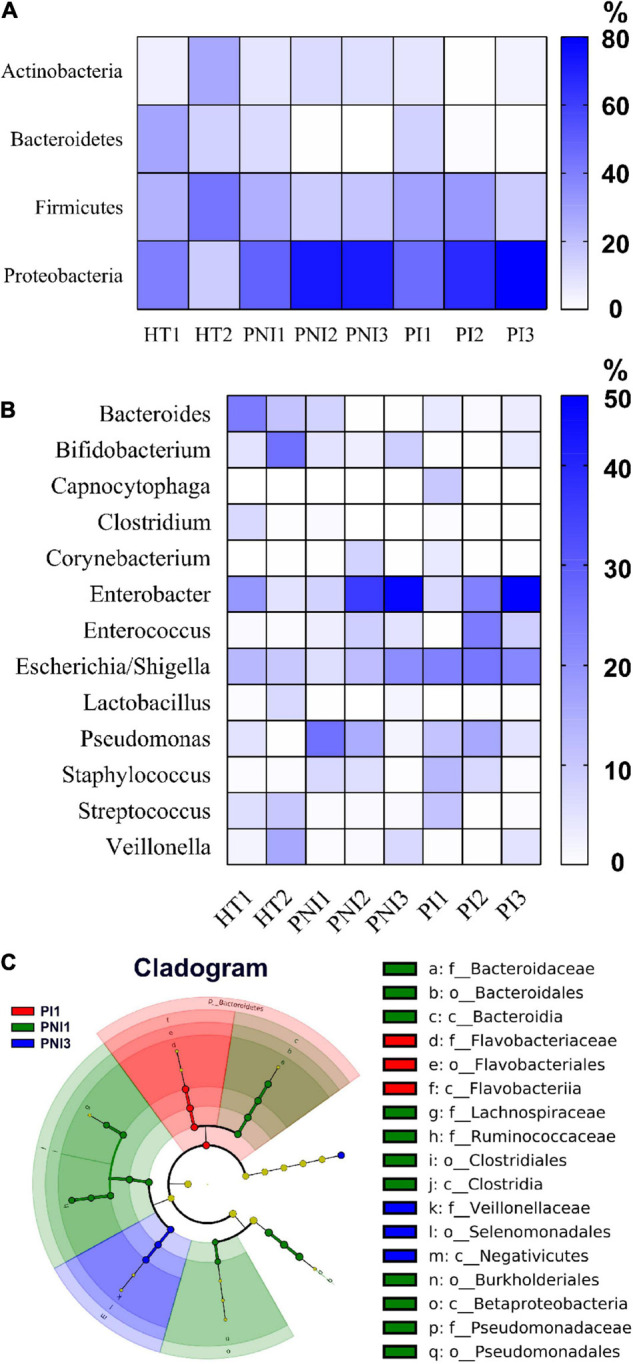
The main compositional proportion of gut microbiota and LefSe analysis. Phylum **(A)** and genus **(B)** levels for each group of participants expressed in heatmaps are shown. **(C)** The cladogram diagram shows the microbial species with significant differences in the three groups. Inside to outside showed the species classification at the level of phylum, class, order, family, and genus. The red, green, and blue nodes in the phylogenetic tree represent microbial species that play an important role in the PI1, PNI1, and PNI3 groups, respectively. Yellow nodes represent species with no significant difference.

Figures 2, 3 show the taxonomy profile and the proportion of major phyla and genera in heatmap, respectively. The phyla of gut microbiota in healthy term infants after birth (HT1) were similar to those in preterm infants after birth (PNI1 and PI1), in which *Proteobacteria* accounted for 40.3–48.8%, followed by *Firmicutes* (23.9–29.5%), *Bacteroidetes* (10.9–28.4%), and *Actinobacteria* (5.2–8.5%). At 4–7 weeks of age, however, the proportion of *Proteobacteria* decreased to 16.3% in HT2 but increased to 72.8 and 80.3% in PNI3 and PI3, respectively. Meanwhile, the proportion of *Firmicutes* increased to 42.6% in HT2 but decreased to 17.9 and 15.7% in PNI3 and PI3, respectively. The trends indicated that gut microbiota evolved toward dysbiosis in preterm infants, especially in the PI group (Shin et al., 2015). In the level of genera, the proportion of GNB (*Enterobacter*, *Escherichia/Shigella*) increased over time among preterm infants, and the magnitude was higher in the PI group than in the PNI group. The organisms with significant differences in the three groups are shown by cladogram in Figure 3. *Bacteroidetes* was the most significant phylum in HT1, *Actinobacteria* in PNI1, and *Proteobacteria* in PI1.

### Impact of Antibiotic Use on Gut Microbiota

All preterm infants in our NICU had antibiotic exposure during hospitalization. The median DOT before the third stool collection of 32 preterm infants was 20.5 days (range, 4–77 days). The fecal samples collected from preterm infants whose DOT was higher than the median were classified into the DOT_>__median_ group and the other into the DOT_<__median_ group. Fecal samples of healthy term infants without antibiotic exposure collected at the fourth week of age (HT2) were used as control. The rates of invasive infections were not significantly different between infants of the DOT_<__median_ group and the DOT_>__median_ group (25.0 vs. 37.5%, *p* = 0.70).

**FIGURE 4:**
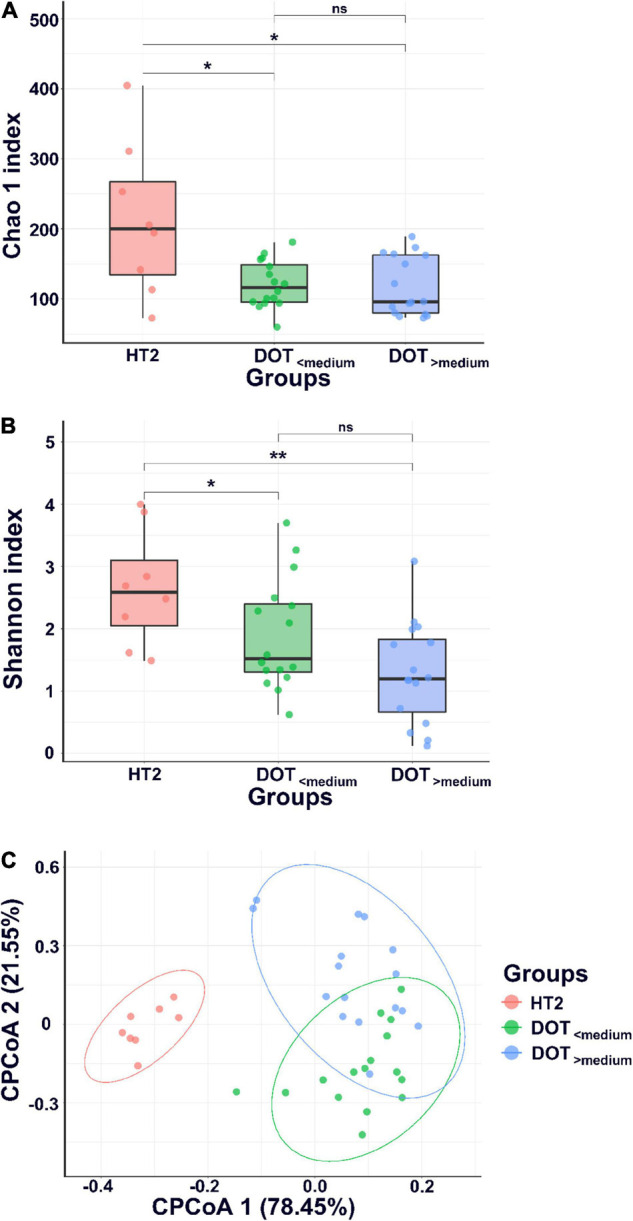
α-diversity and β-diversity of gut microbiota. α-diversity indices, including Chao1 **(A)** and Shannon index **(B)**, and **(C)** β-diversity (CPCoA, covariate adjusted principal coordinates analysis, CPCoA) of gut microbiota of healthy term infants at 4 weeks of age (HT2) and preterm infants with DOT lower than median (DOT_<__median_) and DOT higher than median (DOT_>__median_). *p < 0.05; **p < 0.01; ns, non-significant.

**FIGURE 5:**
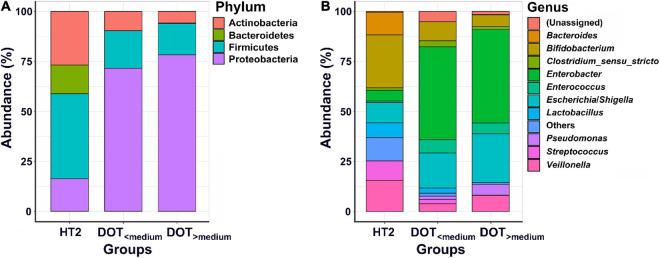
Abundances of bacterial taxa between preterm infants with different antibiotic exposure level. Mean relative abundances of bacterial taxa at phylum **(A)** and genus **(B)** levels in each group of the stool collections. HT2, healthy term infants at 4 weeks of age; DOT_<__median_, preterm infants with DOT lower than median; DOT_>__median_, preterm infants with DOT higher than median.

The microbiome of fecal samples was analyzed to assess the impact of antibiotic use on gut microbiota. The α-diversity (Chao1 and Shannon index) was significantly lower in preterm infants (DOT_<__median_ and DOT_>__median_) than in healthy term infants (HT2) (Figure 4). Analysis of β-diversity also showed significantly different microbiota composition in the gut among HT2, DOT_<__median_, and DOT_>__median_ groups (*p* < 0.001) (Figure 4). The difference of the gut microbiota between healthy terms and preterm infants according to antibiotic exposure is shown in Figure 5 and Supplementary Figure 1. The major phyla in healthy terms were *Firmicutes* (42.6%), *Actinobacteria* (26.8%), *Proteobacteria* (16.3%), and *Bacteroidetes* (14.3%); however, the major phyla in both DOT_<__median_ and DOT_>__median_ were *Proteobacteria* (71.7 and 78.3%, respectively) and *Firmicutes* (18.7 and 15.7%, respectively), while the proportion of *Bacteroidetes* was low in both (Figure 5). In the genus level, the top five predominant genera in healthy terms were *Bifidobacterium* (26.5%), *Veillonella* (15.6%), *Bacteroides* (11.2%), *Escherichia/Shigella* (10.2%), and *Streptococcus* (9.7%), while in DOT_< median_ and DOT_>__median_, the predominant genera were *Enterobacter* (46.3 and 46.7%, respectively), *Escherichia/Shigella* (17.7 and 24.4%, respectively), and *Bifidobacterium* (9.5 and 5.7%, respectively). Gut microbiome in the DOT_>__median_ group was composed of significantly more bacteria like genera *Enterobacter* and *Pseudomonas* than other groups (Supplementary Figure 1). The genus *Bifidobacterium* accounted for 26.5, 9.5, and 5.7% in healthy terms, DOT_<__median_, and DOT_>__median_, respectively. The genus *Lactobacillus* accounted for 7.4, 2.5, and <0.1% in HT2, DOT_<__median_, and DOT_>__median_, respectively. Thus, prolonged exposure to antibiotics did not reduce the proportion of bacteria in the genera *Enterobacter* and *Pseudomonas* but reduced beneficial bacteria, such as *Bifidobacterium* and *Lactobacillus*, in the gut.

### Pathogenic Bacterial Strains Identified in Gut Microbiota

The proportions of the bacterial species identical to the causative pathogens for sepsis in gut microbiota are summarized in Table 2 and Supplementary Figures 2–5, and the results of stool PCR are shown in Table 3. Bacterial species identical to the causative pathogens has been predominant in the gut microbiota of three premature infants with sepsis (C08, C28, and C44). Patient C08 developed LOS by *Klebsiella pneumoniae* between the second and third stool collection, and consistently, the proportion of *Klebsiella* genus increased from 43.9% in the first stool sample to 82.4 and 93.3% in the second and third stool samples, respectively (Table 2 and Supplementary Figure 2, *Klebsiella*). The pathogenic *K. pneumoniae* was only detected by PCR in the second and third stool samples, suggesting that *K. pneumoniae* being present in the first sample was likely not pathogenic (Table 3). The mother of patient C44 had chorioamnionitis by *Escherichia coli* (data not shown), and patient C44 developed EOS by *E. coli* before the first stool collection. The microbiota showed that the *Escherichia* genus accounted for 99.8, 1.8, and 44.9% in the first, second, and third stool samples, respectively (Table 2 and Supplementary Figure 3, *Escherichia*). The pathogenic *E. coli* was indeed detected in the first fecal sample. After antibiotic treatment, the *E. coli* disappeared in the second stool sample; however, it reappeared in the third stool sample (Table 3). Patient C22 had external ventricular drainage (EVD) for post-hemorrhagic hydrocephalus and developed LOS and EVD shunt infection by *S. aureus*. The microbiota in the genus level showed that *Staphylococcus* only accounted for 0.3, 0.3, and 2.4% in the three stool samples (Table 2 and Supplementary Figure 4, *Staphylococcus*). The PCR confirmed that pathogenic *S. aureus* was present in the second and third fecal samples (Table 3). Patient C28 developed LOS by *S. epidermidis* after third stool collection. S*taphylococcus* in gut microbial composition was 93.4, 1.1, and 0.1% in the three fecal samples (Table 2 and Supplementary Figure 5, *Staphylococcus*); however, PCR confirmed the presence of pathogenic *S. epidermidis* in the second stool collection (Table 3).

**TABLE 3:** PCR to identify bacterial pathogens in fecal samples collected from the preterm infants with sepsis.

**Case/Type**	**Bacterial isolate^a^**		**1st fecal sample^b^**		**2nd fecal sample**		**3rd fecal sample**		**Fecal samples from healthy neonates^c^**	
	**P1**	**P2**	**P1**	**P2**	**P1**	**P2**	**P1**	**P2**	**P1**	**P2**
C08/LOS	+	+	-	-	+	+	+	+	-	-
C44/EOS	+	+	+	+	-	-	+	+	-	-
C06-2/LOS	+	+	-	-	-	-	-	-	-	-
C42/LOS	+	+	-	-	-	-	-	-	-	-
C06-1/LOS	+	+	-	-	-	-	-	-	-	-
C03/VLOS	+	+	-	-	-	-	-	-	-	-
C19/LOS	+	+	-	-	-	-	-	-	-	-
C22/LOS	+	+	+	-	+	+	+	+	+^d^	-
C28/LOS	+	+	-	-	+	+	-	-	-	-
C34/LOS	+	+	-	-	-	-	-	-	-	-
C38-1/VLOS	+	+	-	-	-	-	-	-	-	-
C38-2/VLOS	+	+	-	-	-	-	-	-	-	-

*Two unique primers P1 and P2 were used to recognize specific sequence of each bacterial isolate in fecal samples. The details of primers are listed in Supplementary Table 1.*

*EOS, early-onset sepsis; LOS, late-onset sepsis; VLOS, very-late-onset sepsis.*

*^a^Isolated bacterial strains were used as positive controls in the PCR assay.*

*^b^1st, 2nd, and 3rd fecal samples from the preterm infants were collected within 1st, 4th, and 7th week after birth, respectively.*

*^c^Randomly picked three fecal samples from healthy term infants as negative controls in the PCR assay.*

*^d^Positive only in one of the three randomly picked fecal samples from healthy term infants.*

### Results of Strain-Specific PCR

We designed strain-specific primers using genome sequences of the bacterial isolates and performed PCR upon the stool samples to track the dynamic change of the pathogens. The results clearly distinguished the pathogenic clone from the colonizing bacteria of the same species. Colonization of pathogenic bacteria in the gut of patients C08, C22, and C28 with LOS and of patient C44 with EOS was confirmed (Table 3). During the study period, there was an outbreak of *B. cereus* sepsis in NICU. *B. cereus* was confirmed from contaminated linen (data not shown). The present study found no *B. cereus* in the stool samples of patient C42, indicating that *B. cereus* was derived from the environment (Table 3).

PCR with strain-specific primers also examined whether the pathogenic bacteria of the same genotype in different patients were identical strains or not. MLST revealed that *S*. *epidermidis* ST35 contributed to infections in both patients C03 and C38 (first infection), and *S. epidermidis* ST59 to those in patients C28 and C38 (second infection). The results of PCR indicated that *S. epidermidis* ST59 from patients C28 and C38 (second CNS infection) who had hydrocephalus with VPS infection were identical because both were identified by the same strain-specific primers (Table 3). Notably, the two infections occurred nearly 5 months apart, and *S. epidermidis* ST59 was not detected in any fecal sample of patient C38. Furthermore, PCR confirmed that *S. epidermidis* ST35 from patients C03 and C38 (first CNS infection) belonged to different bacterial clones because strain-specific primers only recognized the bacterial isolates these primers were derived from (data not shown).

## Discussion

The development of gut microbiota is generally believed to begin from birth, though some studies detected microorganisms in amniotic fluid, placenta samples, and meconium (Aagaard et al., 2014; Collado et al., 2016). In this study, we found that preterm infants and term infants had similar gut microbiota composition after birth in that the most common phyla in both are *Proteobacteria*, *Bacteroidetes*, *Firmicutes*, and *Actinobacteria*. However, unlike the increase in *Firmicutes* and *Actinobacteria* in term infants, *Proteobacteria* in preterm infants increased significantly in the next few weeks after birth. This phenomenon was found in previous reports that gut microbiota of preterm infants was quickly dominated by *Proteobacteria* species within the first week of life and maintained at high levels throughout the first month of life (Mshvildadze et al., 2008; Morowitz et al., 2011; La Rosa et al., 2014; Thursby and Juge, 2017). It is still unknown whether and when preterm infants will eventually follow a similar developmental pattern if they “catch up” to term infants in postnatal age (Gibson et al., 2015). We also found in this study that the gut in infected preterm infants carried more potentially pathogenic bacteria and less beneficial bacteria than their un-infected counterparts in the microbiota composition.

The gut microbiota in newborns in advance of neonatal sepsis was different depending on timing and areas studied (Taft et al., 2014). Earlier literatures have shown that the gut microbiota with a low diversity in part due to prolonged antibiotic use may be associated with sepsis (Madan et al., 2012; Mai et al., 2013; Graspeuntner et al., 2019). Gut microbiota of infants treated with antibiotics in the first 2 days of life had significantly higher proportions of the phyla of *Proteobacteria* and significantly lower proportion of *Actinobacteria* (mainly genera of *Bifidobacterium* and *Lactobacillus*) (Fouhy et al., 2012). These notions are supported by the findings in the present study: VLBW preterm infants with invasive infection had lower diversity in microbiota after birth, and the microbial diversity was inversely correlated to the duration of antibiotic exposure. Furthermore, the prolonged antibiotic use significantly reduced beneficial *Bifidobacterium* and *Lactobacillus* in the gut. Thus, gut dysbiosis is a major risk factor for the development of neonatal sepsis, and the early change of gut microbiota can be used as a marker to predict or prevent sepsis.

The organisms for EOS are typically colonizers of the maternal genitourinary tract, leading to contamination of the amniotic fluid, placenta, cervix, or vaginal canal, and then ascending to cause an intra-amniotic infection (Shah and Padbury, 2014). The pathogenesis of LOS and VLOS is still debated and is hypothesized that pathogenic bacteria may invade into bloodstream from the environment or from previously colonized bacteria in the gut. In our study, among the 12 episodes of sepsis, the only EOS with *E. coli* infection resulted from the mother’s chorioamnionitis. The other eight LOS and three VLOS were mostly caused by *Staphylococcus* species, consistent with the previous observation that the predominant organisms among VLBW neonates are coagulase-negative staphylococci, which can occur in association with medical device or occur *de novo* (Bizzarro et al., 2005).

This study shed new insights about the mechanism of neonatal sepsis and provided possible prevention strategies. First, PCR with strain-specific primers confirmed the colonization of the bacterial pathogens in the gut of three patients before they developed sepsis, suggesting the translocation of bacteria from the gut to the bloodstream. Second, two patients C28 and C38 (second CNS infections) were caused by the same bacterial strain, but the strain was not detected in stools of C38, hinting that the latter infection may be from the environment. The two infections occurred nearly 5 months apart, suggesting that the bacterial pathogen may persist in the environment or colonize in non-infected hospitalized infants or medical staff. There were similar reports that gut colonization is the major risk factor for horizontal dissemination and subsequent infection to surrounding patients (Martin et al., 2016; Gorrie et al., 2017). Therefore, infection control measures and environmental surveillance after an invasive infection occurs should be reinforced in the NICU. Third, despite antibiotic treatment, the bacterial strains persisted in stools of patients C08 (EOS) and C44 (LOS), increasing the risk of horizontal transmission. The pathogenic bacteria persistently colonized in the gut, which highlighted the importance of hand washing to prevent bacterial transmission in the NICU. Fourth, using PCR with strain-specific primers, we distinguished two infections caused by different bacterial strains with the same genotype. The molecular testing methods are helpful for the investigation of the clusters of LOS in the NICU. Our study demonstrated that causative organisms for LOS in hospitalized preterm infants may originate either from colonized bacteria in the gut or be acquired horizontally from the environment.

The strength of our study is to provide information of gut microbiota compositions between term and preterm infants with or without sepsis. In addition to genomic analysis, we also used specific PCR to prove the presence of causative pathogens in the gut and to distinguish bacteria with the same genotype. There are also some weaknesses: first, only three fecal collections for each patient limited the investigation of the duration of bacterial colonization in neonates; second, the enrolled VLBW preterm infants composed only a small percentage of the hospitalized patients, thereby limiting a comprehensive investigation on causative organisms and the modes of dissemination; and third, considering that healthy term infants were in a mature and stable state, we did not collect their stool samples for examination in the seventh weeks of life. Nevertheless, the study provided evidence demonstrating the transmission dynamics of bacterial pathogens for neonatal sepsis in preterm infants less than 3 months of age.

## Conclusion

The study revealed that VLBW preterm infants with gut dysbiosis are at risk for neonatal sepsis, and the causative pathogens may be bacteria that previously colonize in the gut or be acquired horizontally from the environment. Prolonged exposure to antibiotics is a significant cause for gut dysbiosis in preterm infants. Microbiota analysis suggests the need for interventions targeting the gut microbiome to prevent dysbiosis and sepsis in VLBW preterm infants.

## Materials and Methods

### Study Design and Sample Collection

This prospective cohort study was approved by the Institutional Review Board of Chang Gung Memorial Hospital (201702153B0). Preterm infants with birth weight between 500 and 1,500 g and no congenital gastrointestinal anomaly were enrolled after obtaining consent within 72 h of age between August 2018 and October 2019. Fecal samples in the first, fourth, and seventh weeks of life were collected. All participants were followed up for 6 months. Those who had clinical symptoms of infection and bacteria yielded from sterile sites, such as blood or cerebral spinal fluid were defined as having invasive infections or sepsis (Shane et al., 2017). Clinical information about pregnancy complications, perinatal conditions, and any postnatal infections was also recorded. We also collected stool samples from healthy term infants as controls within the first and fourth weeks of age during the study period. All stool samples were analyzed with 16S rRNA gene sequencing to investigate the intestinal flora. All invasive bacterial isolates were subjected to genome sequencing. According to the genomic sequences, strain-specific primers were designed for each bacterial isolate, and PCR was done to check whether the invasive bacteria derive from the gut. In addition, the amount of antibiotics for each patient was measured by days of therapy (DOTs) and was calculated as the sum of the total number of days of all used antibiotics (Momattin et al., 2018).

### Fecal Sample Processing

Stool samples were collected from diapers using the Longsee Fecalpro Kit (Longsee Medical Technology Co., China). Genomic DNA extraction was performed using the QIAamp PowerFecal DNA Kit (Qiagen, United States) according to the instructions of the manufacturer. About 250 μg of the sample was added to 750 μl of PowerBead Solution and 60 μl of Solution C1, and the tube was heated at 65°C for 10 min. Then, the mixture was vortexed using a PowerLyser Homogenizer for 10 min at 1,000 RPM. The DNA was extracted and washed, eluted using nuclease-free water according to the QIAamp PowerFecal DNA Kit handbook. The extracted DNA was stored at -80°C. The concentrations and qualities of the purified DNA were determined by Qubit 4 Fluorometer with high-sensitivity dsDNA assay (Thermo Fisher Scientific).

### Whole Genome Sequencing of Bacterial Isolates

The genomes of the cultured bacteria from patients with invasive infections were sequenced with Illumina Miseq platform (Illumina, San Diego, CA, United States). The short reads generated were *de novo* assembled into contigs using SPAdes version 3.11.1 (Bankevich et al., 2012). The assembled genome sequences have been submitted to the NCBI GenBank database under the accession number PRJNA674911.^1^ The multilocus sequencing typing (MLST) was performed by the online database BacWGSTdb (Ruan and Feng, 2016). For each isolate, the genome was compared against the representative genomes in BacWGSTdb through the Cross_match program. The derived sequence-type specific fragments were used for designing specific primers for subsequent PCR confirmation.

The MLST was performed by using the protocol in the online database BacWGSTdb (Ruan and Feng, 2016). For each isolate, the genome was compared against the representative genomes in BacWGSTdb through the Cross_match program. The derived sequence-type specific fragments were used to design specific primers for subsequent PCR confirmation (Supplementary Table 1). For each strain, two pairs of primers targeting two genes were designed. To prove the presence of cultured pathogenic bacterial strains in feces, two unique primers to recognize the specific sequence of pathogenic bacterial strains were designed for each cultured pathogenic bacterium. Each fecal sample was processed for PCR to confirm the presence of the causative bacteria in the stool.

### Unique Primers

The procedures for primer designing include the following: (1) choose a DNA fragment that does not come from a general plasmid region, determined by means of NCBI BLASTX with a database of non-redundant protein sequence and with a specific organism at https://blast.ncbi.nlm.nih.gov/Blast.cgi; (2) select a sequence (150–1,500 bps) of the chosen DNA fragment that has a unique single nucleotide polymorphism aligned with other sequences of sources at both ends (Ruan and Feng, 2016). The two end sequences are designed as a set of PCR primers with a size of 20--25 bps and 40--60% GC content by means of Nucleotide BLAST^2^ or Primer 3.^3^ Primers used in this study are listed in Supplementary Table 1. Each fecal sample was processed for PCR with unique primers to find pathogenic bacteria strains. Total RNA was reversely transcribed, and the subsequent cDNA samples were subjected to real-time PCR using SYBR Green (Vazyme, China) and the primer sets.

### Bioinformatic Processing

The bacterial 16S rRNA gene amplicon was constructed based on the hypervariable V3–V4 region (Klindworth et al., 2013) and sequenced using the MiSeq System (Illumina, United States). The 16S rRNA gene amplicon library sequencing reads were initially demultiplexed using MiSeq Reporter v2.6 according to sample barcodes. Raw reads were performed using Usearch (V.11)^4^ for pairing reads, quality filtering, and operational taxonomic unit (OTU) clustering. The RDP training set (v16) database was used as a reference set for final taxonomic assignments performed using the SINTAX classifier. The α-diversity (e.g., Chao index and Shannon index) and β-diversity measurements were calculated. Finally, we used linear discriminant analysis (LDA) of effect size (LEfSe) to determine the taxonomy that most likely explains the differences between groups (Segata et al., 2011).

### Statistics Analysis

The clinical characteristics were analyzed using Student’s *t*-test and chi-square test. The relative abundance of bacteria and alpha diversity indices were compared and displayed by GraphPad Prism 8 (GraphPad Software, Inc., La Jolla, CA, United States). Between-group comparisons were performed with a random forest for two groups. We performed multivariate logistic regression analysis on the bacteria markers and used the statistical logistic forward method to produce a panel to identify case patients. Statistical analyses were performed using the IBM SPSS Statistics software, version 25.0 (IBM Inc., Chicago, IL, United States). The tests were two-sided, and a *p* < 0.05 was considered statistically significant.

## Data Availability Statement

The assembled genome sequences have been submitted to the NCBI GenBank database under the accession number PRJNA674911 (www.ncbi.nlm.nih.gov/bioproject/PRJNA674911/).

## Ethics Statement

This cohort study was approved by the Institutional Review Board of Chang Gung Memorial Hospital (201702153B0). Written informed consent from the participants’ legal guardian/next of kin was not required to participate in this study in accordance with the national legislation and the institutional requirements.

## Author Contributions

C-CL, YF, and C-HC: conception and design of the study. YF, C-CL, RL, C-LC, and Y-LZ: implementation and data collection. C-CL, YF, Y-MY, and C-HC: analysis and interpretation of the data. C-CL, YF, Y-MY, RL, and C-HC: writing and critical review of the manuscript. All authors contributed to the article and approved the submitted version.

## Conflict of Interest

The authors declare that the research was conducted in the absence of any commercial or financial relationships that could be construed as a potential conflict of interest.

## Publisher’s Note

All claims expressed in this article are solely those of the authors and do not necessarily represent those of their affiliated organizations, or those of the publisher, the editors and the reviewers. Any product that may be evaluated in this article, or claim that may be made by its manufacturer, is not guaranteed or endorsed by the publisher.

## Footnotes

1
https://www.ncbi.nlm.nih.gov/bioproject/PRJNA674911/

2
https://blast.ncbi.nlm.nih.gov/Blast.cgi

3
http://bioinfo.ut.ee/primer3-0.4.0/

4
https://drive5.com/
