# Supplementary material for: Gut Dysbiosis, Bacterial Colonization and Translocation, and Neonatal Sepsis in Very-Low-Birth-Weight Preterm Infants
Source: Front Microbiol. 2021 Oct 7;12:746111. doi: 10.3389/fmicb.2021.746111 (PMC8529156; doi:10.3389/fmicb.2021.746111)
Supplement: Supplementary file 1 [file Table_1.docx]

**SUPPLEMENTARY TABLE 1 |** List of strain-specific primers for bacterial isolates of neonatal sepsis.

| **Primers** | **Case, Bacterial species** | **Primer direction** | **Sequence (5' to 3')** | **Gene** | **Size (bp)** |
| --- | --- | --- | --- | --- | --- |
| **1** | C42,  *Bacillus cereus* | 1^st^ Forward | CAGTGGAAAAACACTAAAAAA | DNA-3-methyladenine glycosylase | 729 |
|  |  | 1^st^ Reverse | CCACTATTTACAAATTGTAAC |  |  |
|  |  | 2^nd^ Forward | TAAACCACGAGTAGAGGATTA | YafY family transcriptional regulator | 664 |
|  |  | 2^nd^ Reverse | ATAGAGAGATAGAACCTATGG |  |  |
| **2** | C03,  *Staphylococcus epidermidis* | 1^st^ Forward | TGGTGATATTTCTAATGGTGTA | Virulence-associated protein E | 1123 |
|  |  | 1^st^ Reverse | CCCATGTTTTAGATTCAATCAT |  |  |
|  |  | 2^nd^ Forward | AATTAATAGAGCATCCCTCAC | CDP-glycerol: glycerophosphate glycerophosphotransferase | 287 |
|  |  | 2^nd^ Reverse | CTATGAACCATAACACTACA |  |  |
| **3** | C06-1,  *Staphylococcus capitis* | 1^st^ Forward | ACGACCCAACTCATCATGAAC | Acyl-CoA thioester hydrolase | 194 |
|  |  | 1^st^ Reverse | TAAGCGCCCCAAGCTAATATC |  |  |
|  |  | 2^nd^ Forward | TCAAGAAGGACAAGCAGATGG | SGNH/GDSL hydrolase family protein | 452 |
|  |  | 2^nd^ Reverse | CGTATTCAGTATCGCCGACAT |  |  |
| **4** | C06-2,  *Serratia liquefaciens* | 1^st^ Forward | ATGCTGGAGGAAGAATGACAC | Transposase | 330 |
|  |  | 1^st^ Reverse | GCGTTACAGCAGATTTGAAGC |  |  |
|  |  | 2^nd^ Forward | TGCATCTGGTTAGGTTTACCG | Tyrosine-type recombinase/integrase | 387 |
|  |  | 2^nd^ Reverse | AGACGAGATTCCAAGACAGCA |  |  |
| **5** | C08,  *Klebsiella pneumoniae* | 1^st^ Forward | GTGTCATCCGGTAATGATTGG | YfbU family protein | 715 |
|  |  | 1^st^ Reverse | CGTGAACGCAGAGAACCTAAG |  |  |
|  |  | 2^nd^ Forward | AAGTCCGCAGGATTAGCTTTC | Glutathione synthase/Ribosomal protein S6 modification enzyme | 324 |
|  |  | 2^nd^ Reverse | AAATACTCCAGGGCGTAGCAT |  |  |
| **6** | C19,  *Staphylococcus capitis* | 1^st^ Forward | CATGGTAGGAAGCACATACCG | Helix-turn-helix transcriptional regulator | 693 |
|  |  | 1^st^ Reverse | CGATCTGTCGTGTGTTCATGT |  |  |
|  |  | 2^nd^ Forward | CTTCCCCAGCACCTCTTTTAC | DUF1381 domain-containing protein | 835 |
|  |  | 2^nd^ Reverse | AATTGGAGCGTGGTAGTGATG |  |  |
| **7** | C22,  *Staphylococcus aureus* | 1^st^ Forward | AACCTAAACGAAACCCGCCTA | DNA mismatch repair protein MutH | 765 |
|  |  | 1^st^ Reverse | AACCATTCCCGGAAGTTGAAG |  |  |
|  |  | 2^nd^ Forward | TGACTGGGATGGATTTTGTGA | RES family NAD+ phosphorylase | 501 |
|  |  | 2^nd^ Reverse | ATTCCTTGTTCATCGCTTGGA |  |  |
| **8** | C28, C38-2  *Staphylococcus epidermidis* | 1^st^ Forward | TACTCAAGGGAATGGCCAAGA | Ser-Asp rich fibrinogen-binding protein | 733 |
|  |  | 1^st^ Reverse | TGTTGTTGGTTCACCACGTTC |  |  |
|  |  | 2^nd^ Forward | TAAGTTTGGCGAGCTTTGGTG | HNH endonuclease | 720 |
|  |  | 2^nd^ Reverse | GACCGCCATGTCAAGGTTTTA |  |  |
| **9** | C34,  *Staphylococcus capitis* | 1^st^ Forward | TGTTCCAAACGACAACGTCAC | SH3 domain-containing protein | 677 |
|  |  | 1^st^ Reverse | ATCGTTTCGGCGATAATCCTT |  |  |
|  |  | 2^nd^ Forward | GCAATAAATGCCCTCATTCCA | Dam family site-specific DNA-(adenine-N6)-methyltransferase | 563 |
|  |  | 2^nd^ Reverse | TCGTGGAATCCTCCTTTTTGA |  |  |
| **10** | C44  *Escherichia coli* | 1^st^ Forward | TGAACGCCCTCAAGTTCATCT | Virulence-associated protein E | 779 |
|  |  | 1^st^ Reverse | AGATCATTGGTTGCCTGTTGC |  |  |
|  |  | 2^nd^ Forward | CCGTGGATTGGTTGAAGGTAA | AntA/AntB anti-repressor family protein | 583 |
|  |  | 2^nd^ Reverse | CCGTATGCAGGTCCATTTGTT |  |  |
| **11** | C38-1  *Staphylococcus epidermidis* | 1^st^ Forward | GCTTGCCTGAGCATCCATATC | ParA family protein | 693 |
|  |  | 1^st^ Reverse | GATCCCTCCGCATAGCTCTTT |  |  |
|  |  | 2^nd^ Forward | AAAAACAGTCAGCGTGGCACT | AAA family ATPase | 582 |
|  |  | 2^nd^ Reverse | CGAACCATTTAGCCAAATCCA |  |  |
