# Supplementary material for: Gut Dysbiosis, Bacterial Colonization and Translocation, and Neonatal Sepsis in Very-Low-Birth-Weight Preterm Infants
Source: Front Microbiol. 2021 Oct 7;12:746111. doi: 10.3389/fmicb.2021.746111 (PMC8529156; doi:10.3389/fmicb.2021.746111)
Supplement: Supplementary file 2 [file Data_Sheet_1.DOCX]

**SUPPLEMENTARY FIGURES**

**Supplementary Figure 1**


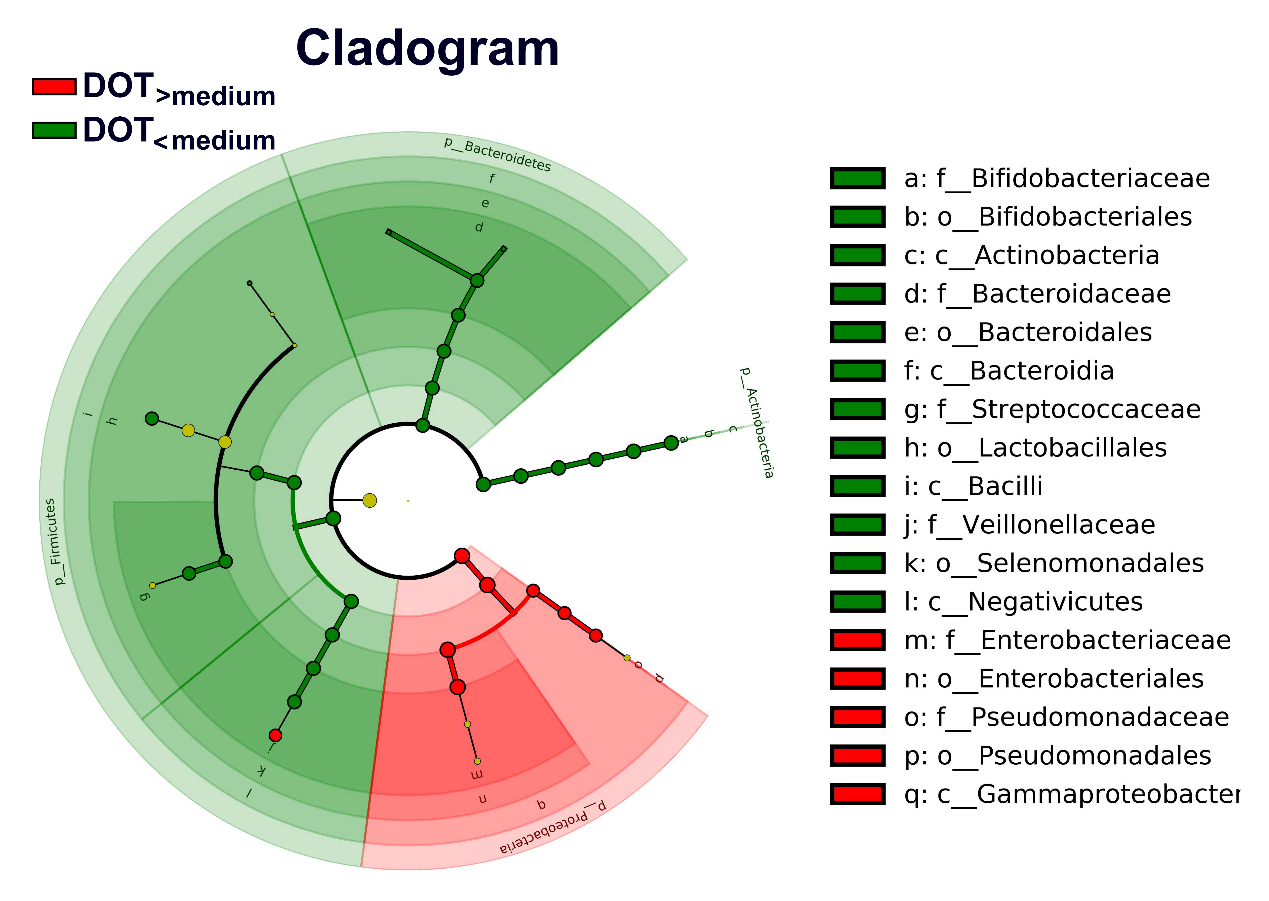


**SUPPLEMENTARY FIGURE 1 |** LefSe analysis. The cladogram diagram shows the microbial species with significant differences in the three groups (see **Figures 4** and **5**). The green and red nodes in the phylogenetic tree represent microbial species that play an important role in the DOT_<median_ and DOT_>median_ groups, respectively. Yellow nodes represent species with no significant difference.

**Supplementary Figure 2**

1. Stool collected within first week of age.


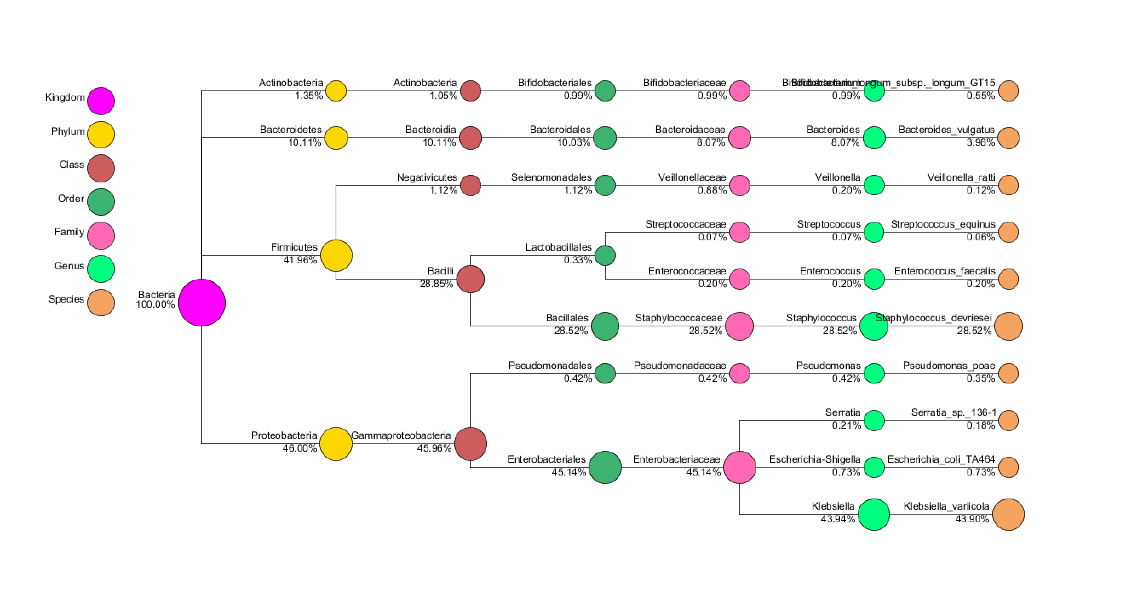


1. Stool collected at 4 weeks of age.


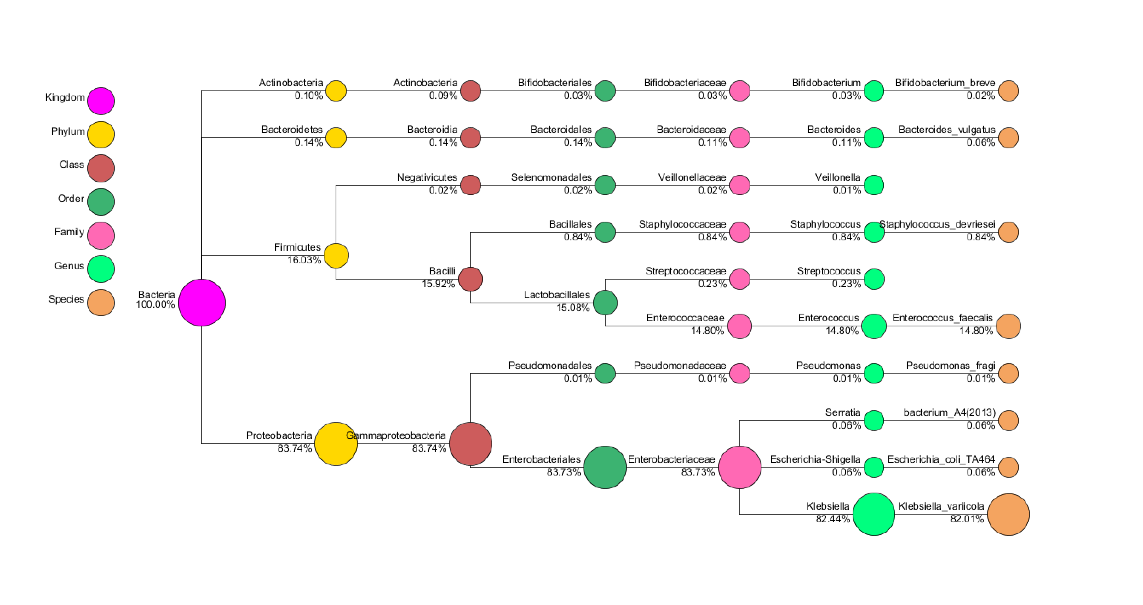


1. Stool collected at 7 weeks of age.


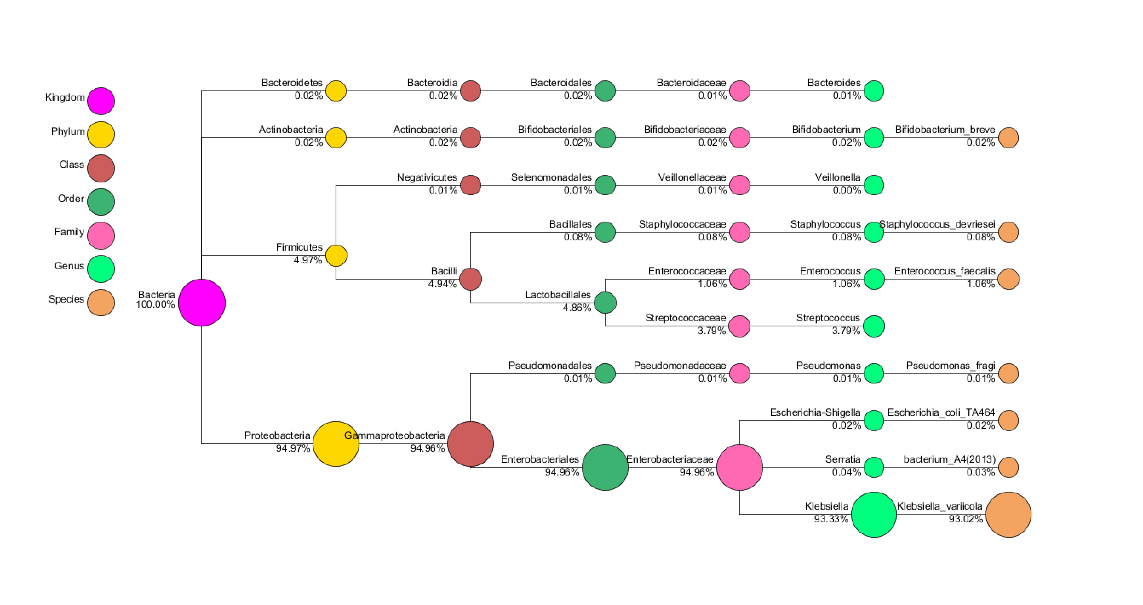


**SUPPLEMENTARY FIGURE 2 |** Top 10 dominant bacteria of case 08. Stool samples were collected at 1^st^ week (**A**), 4^th^ week (**B**), and 7^th^ week (**C**) after birth in patient C08.

**Supplementary Figure 3**

1. Stool collected within first week of age.


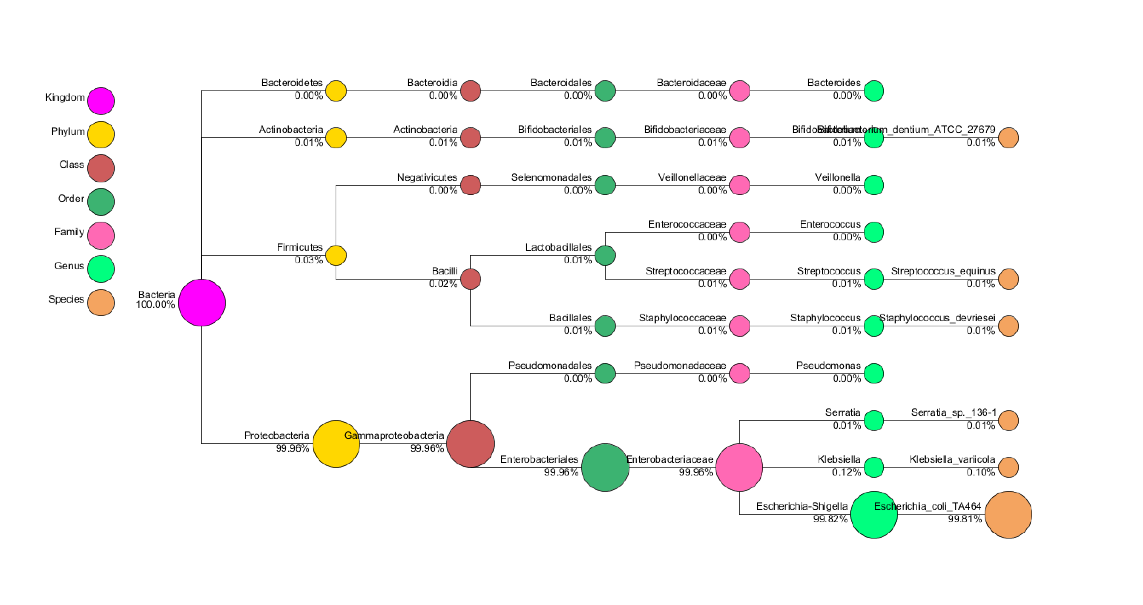


1. Stool collected at 4 weeks of age.


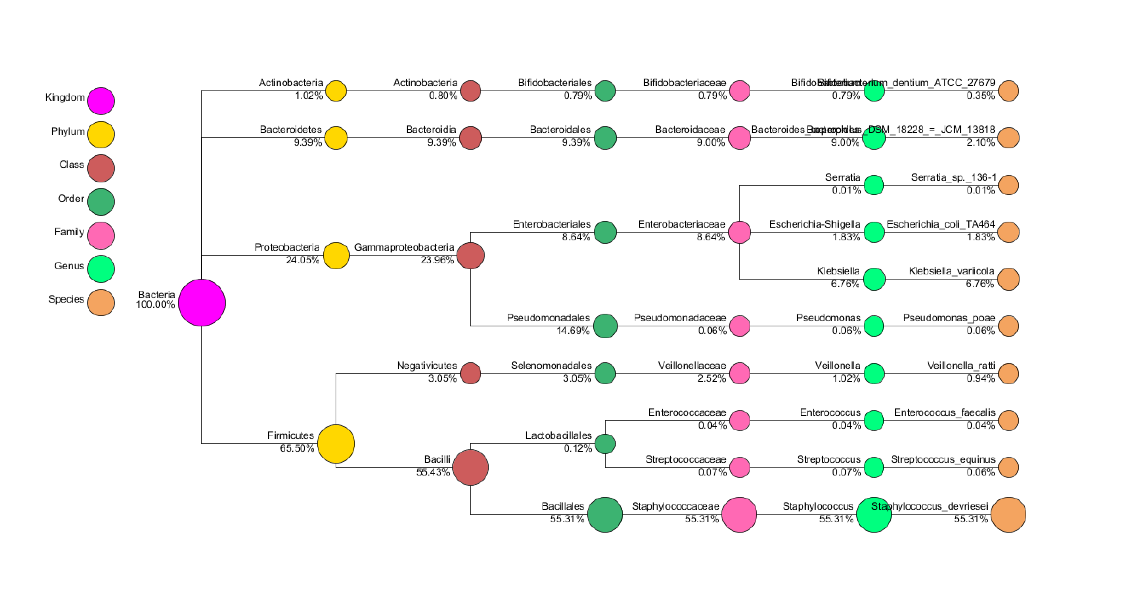


1. Stool collected at 7 weeks of age.


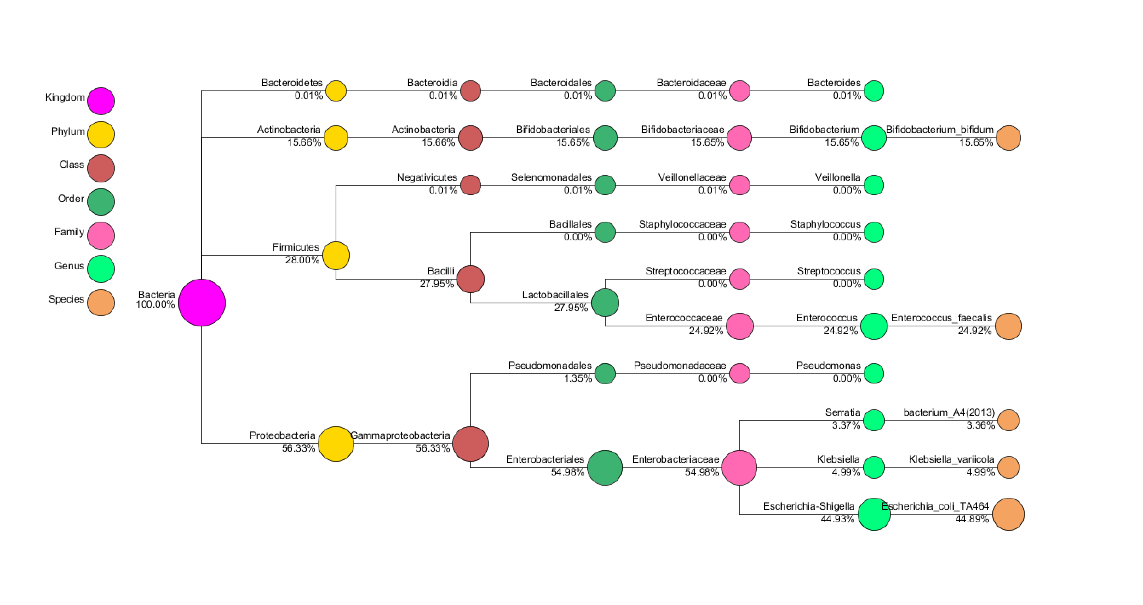


**SUPPLEMENTARY FIGURE 3 |** Top 10 dominant bacteria of case 44. Stool samples were collected at 1^st^ week (**A**), 4^th^ week (**B**), and 7^th^ week (**C**) after birth in patient C44.

**Supplementary Figure 4**

1. Stool collected within first week of age.


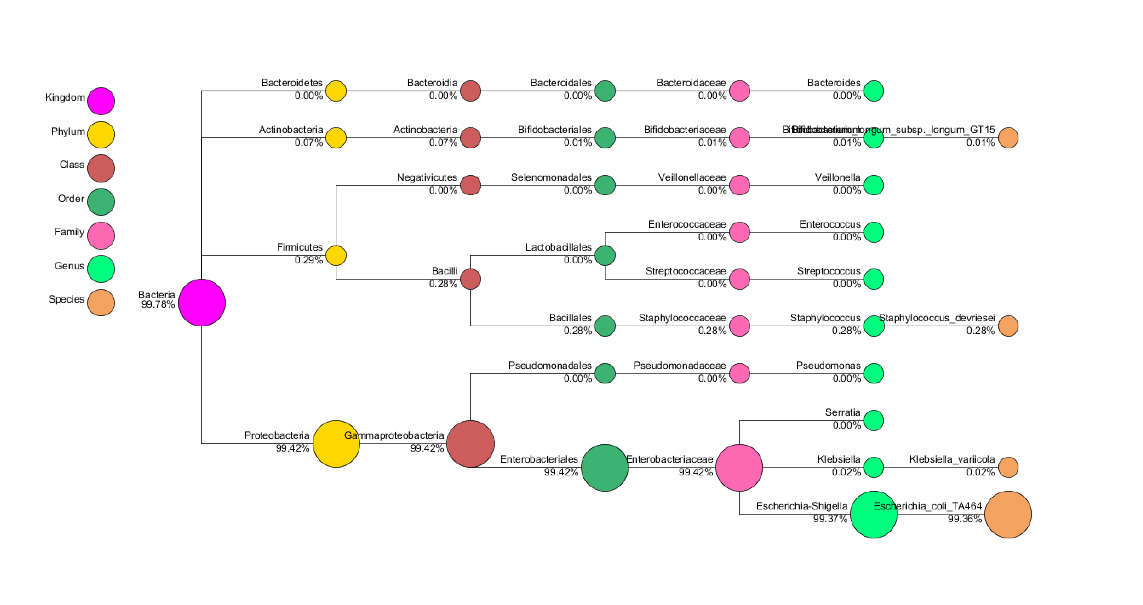


1. Stool collected at 4 weeks of age.


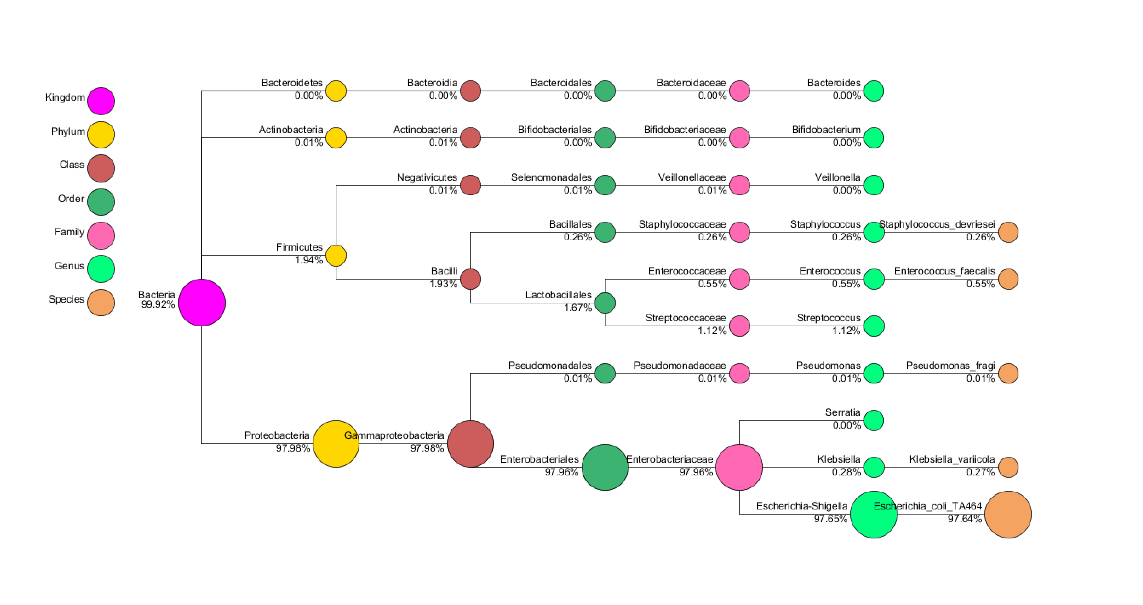


1. Stool collected at 7 weeks of age.


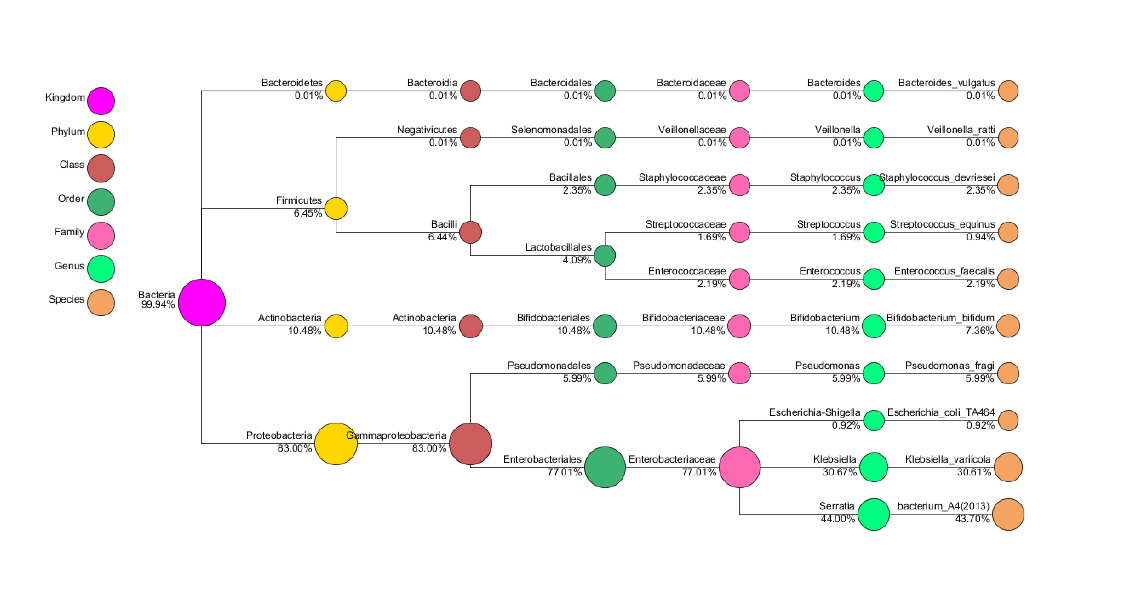


**SUPPLEMENTARY FIGURE 4 |** Top 10 dominant bacteria of case 22. Stool samples were collected at 1^st^ week (**A**), 4^th^ week (**B**), and 7^th^ week (**C**) after birth in patient C22.

**Supplementary Figure 5**

1. Stool collected within first week of age.


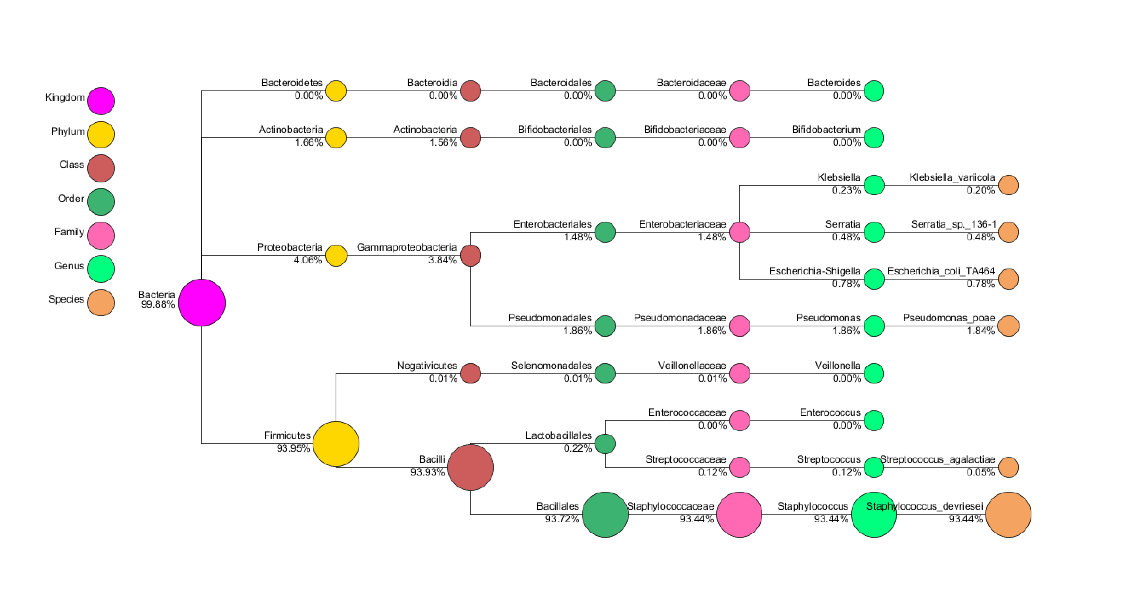


1. Stool collected at 4 weeks of age.


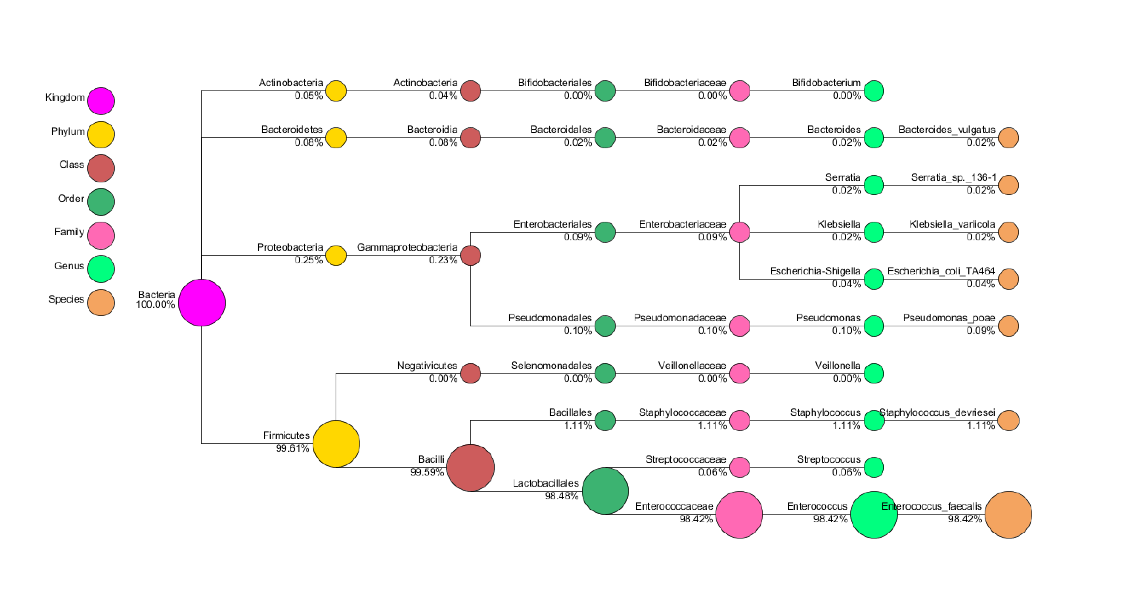


1. Stool collected at 7 weeks of age.


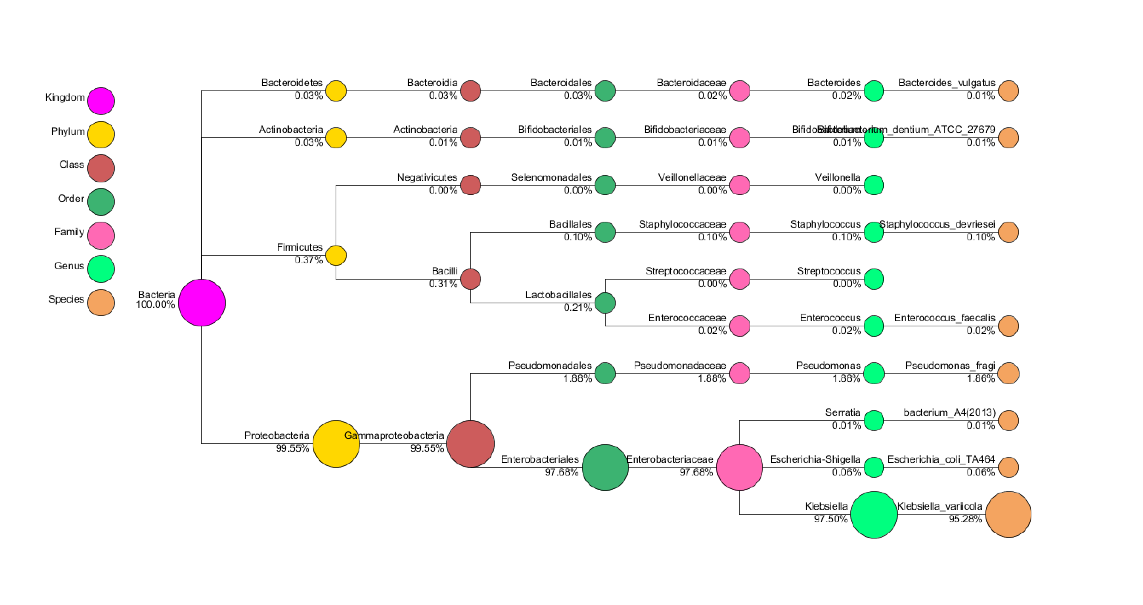


**SUPPLEMENTARY FIGURE 5 |** Top 10 dominant bacteria of case 28. Stool samples were collected at 1^st^ week (**A**), 4^th^ week (**B**), and 7^th^ week (**C**) after birth in patient C28.
